# Cardiovascular Disease and Chronic Endodontic Infection. Is There an Association? A Systematic Review and Meta-Analysis

**DOI:** 10.3390/ijerph18179111

**Published:** 2021-08-29

**Authors:** Despina Koletsi, Anna Iliadi, Giorgos N. Tzanetakis, Manolis Vavuranakis, Theodore Eliades

**Affiliations:** 1 Clinic of Orthodontics and Pediatric Dentistry, Center of Dental Medicine, University of Zurich, CH-8032 Zurich, Switzerland; theodore.eliades@zzm.uzh.ch; 2 Department of Dental Biomaterials, School of Dentistry, National and Kapodistrian University of Athens, 10679 Athens, Greece; annaeliades@gmail.com; 3 Department of Endodontics, School of Dentistry, National and Kapodistrian University of Athens, 10679 Athens, Greece; gtzanet@dent.uoa.gr; 4 First Cardiology Department, National and Kapodistrian University of Athens, Hippocration Hospital, 10679 Athens, Greece; vavouran@otenet.gr

**Keywords:** apical periodontitis, cardiovascular disease, chronic endodontic infection, coronary heart disease, periapical lesion

## Abstract

The aim of the present study was to systematically assess existing evidence on the possible association between chronic endodontic infections and cardiovascular disease (CVD). An electronic database search was implemented until 2 October 2020. The main outcome was risk of CVD diagnosis. Risk of bias was assessed through the ROBINS-I tool, while random effects meta-analyses were conducted. The quality of the evidence was assessed with the Grading of Recommendations Assessment, Development, and Evaluation. Twenty-one studies were eligible for inclusion, while 10 were included in the quantitative synthesis. Risk for CVD diagnosis in patients with chronic endodontic infection was 1.38 times those without infection (RR = 1.38; 95% CIs: 1.06, 1.80; *p* = 0.008). Risk of bias ranged from moderate to serious, while the quality of the evidence was graded as very low. Indications for an identified association between chronic endodontic infection and CVDs do exist; however, they are not grounded on high-quality evidence at present. Further research for an establishment of an association based on temporal sequence of the two entities and on unbiased well-conducted cohort studies would be highly valued.

## 1. Introduction

The global burden of cardiovascular diseases (CVDs), comprising ischemia, ischemic episode, coronary heart disease, stroke, arterial/vascular disease, heart defects, and others, has been widely recognized as of utmost severity and importance for public and community health, and they are also currently identified as the most common non-communicable diseases worldwide. The latest scheming for CVDs impact has revealed a proximal calculation of 330 million years of life lost worldwide, with a corresponding number of 17.8 million deaths [1]. The figures may evidently appear daunting; however, a large number of potentially prognostic factors have been identified and are continuously under close investigation for documentation of association effects with CVDs-related mortality and prediction risk model estimators; efforts to this direction are unceasingly intense, due to the substantial impact of CVDs on everyday life health state and related morbidity [2].

The most common risk factors for CVDs include smoking status, history of diabetes mellitus, blood cholesterol levels, and increased systolic blood pressure [3]. Risk models and confounder standardized calibrated procedures are currently followed in an attempt to provide the most rigorous evidence on the combined fatal and non-fatal events and related risk classification [4]. Interestingly, there has been ongoing research since the start of the millennium, with respect to the identification of potential associations between oral conditions, more specifically inflammation of periodontal tissues and CVDs [5]. This cross-linkage originates form the inherent microbial burden associated with the periodontal disease and its potential for systemic implications [6,7]. Periodontal disease or marginal periodontitis is a well-established inflammatory process of the supporting tooth structures, comprising the periodontal ligament, tooth cementum, and alveolar bone socket, while it affects almost ≈15% of the population in varying degrees. The clinical and radiographic picture of marginal periodontitis is framed under tooth flaring and mobility as well as reduction in the marginal bone support of the teeth in variable levels [5]. Evidence exists on several epidemiologic indicators with regard to periodontitis and CVDs. There is growing evidence for a positive association between marginal periodontitis and coronary heart disease, as confirmed by the latest consensus report of periodontal research [8]. Underlying mechanisms of such an association suggest that oral bacterial species may enter circulation and induce bacteremia [9,10], while the presence of oral bacteria has also been confirmed in atheromatic lesions [11].

On the same grounds, there is growing concern that alternative-origin oral cavity-related chronic inflammatory conditions might be an additional triggering factor for the advent of CVDs. One of the most concerning clinical entities reported is root canal infection and subsequent inflammation of periapical tissues after pulp necrosis described under the term “apical periodontitis”. Apical periodontitis (AP) is an inflammatory disease caused by the establishment of microbial infection within the root canal system of the tooth, resulting in inflammatory periapical tissue response and apically framed bone destruction, as is also evident as a radiolucency in periapical radiographs [12,13]. In turn, this may be related to elevated systemic concentrations of inflammatory mediators or reactive peripheral blood cells, impacting on general cardiovascular health status.

To date, a number of epidemiologic studies have attempted to investigate individual associations between chronic endodontic infections and CVDs; however, strong correlations appear hard to establish due to the inherent risk of bias and threats to the internal validity identified in those studies [14,15,16]. A recent umbrella review [17] has identified four systematic reviews on the topic, which included published studies until four years ago, while a meta-analysis of four studies was performed in only one of those; however, since then, a number of potentially eligible for investigation studies have emerged in the literature [16,18,19], while most importantly, the existing systematic reviews have been graded as critically low to moderate quality, in terms of methodological/reporting inconsistencies followed during the review process [17].

Evidently so, an updated attempt to appraise and synthesize the most recent epidemiologic data in the field is considered timely in order to provide an unequivocal picture of endodontic infections and their association with CVDs, following rigorous and transparent methodology, while also allowing for further insights. In addition, evidence of association, undeniably under the spectrum of causality effects and methodologic limitations of the included studies, might prove a useful guide for future prognostic risk factors to be considered broadly, when estimating individual or integrated effects on CVDs. Therefore, the aim of the present study was to systematically collect and appraise the existing contemporary evidence on the association between chronic endodontic infections and CVDs.

## 2. Materials and Methods

### 2.1. Protocol, Registration, Reporting

The protocol of this study was registered with the Open Science Framework (osf.io/jafgd) [20]. Reporting was conducted in line with the MOOSE guidelines, in view of the anticipated observational design of the potentially eligible for inclusion studies [21,22].

### 2.2. Eligibility Criteria

Eligibility criteria for study selection were structured as follows:

1. Study design: any type of observational study, irrespective of the design, i.e., retrospective cohort, prospective cohort, case-control, cross-sectional.
2. Participants: participants of any age and gender.
3. Condition of interest/exposure: any type of periapical or periradicular lesion indicating infection of the root canal system and inflammation of periapical tissues (also reported as “apical periodontitis”), as confirmed clinically or radiographically/also assessed through medical file records.
4. Outcome: any outcome related to CVDs, incidence or prevalence of the disease, including but not confined to coronary heart disease, stroke, myocardial infraction, arterial/vascular disease.
5. Self-reporting of the disease or the exposure were also considered, but specifically indicated as such, while for cross-sectional study design, the terms exposure/outcome were used interchangeably.
6. Exclusion criteria: case studies, case reports with less than 10 participants.

### 2.3. Search Strategy and Study Selection

Initial electronic searching was conducted within both published and unpublished literature, with no chronologic restriction, or other limits applied. The following Electronic Databases were searched as of May 2020 and updated 2 October 2020: MEDLINE via PubMed, Scopus, Cochrane Central Register of Controlled Trials (CENTRAL), Cochrane Database of Systematic Reviews (CDSR). In addition, gray literature was searched through dissertation sources and in Open Grey, the ClinicalTrials.gov (www.clinicaltrials.gov), and the National Research Register (www.controlled-trials.com). Hand searching of the articles eligible for inclusion was also employed. Keywords included “cardiovascular disease”, “coronary heart disease”, “endodontic infection”, “chronic endodontic infection”, and “apical periodontitis”. The search strategy for PubMed is presented in Appendix A.

### 2.4. Data Collection

Data extraction was employed in pre-piloted standardized forms by two independently working reviewers (DK, AI) not blinded to study origin or author identity. Specifically, information entries were related to study identity, study design, sample size, condition/exposure, outcomes, and further study-specific information. 

### 2.5. Risk of Bias in Individual Studies

Risk of bias assessment was performed independently by one author (DK), and all recordings were confirmed by a second (AI). Any disagreements were settled after consultation with a third author (TE). The ROBINS-I tool was used to assess internal validity of the included studies, in view of the anticipated observational design of the latter [23].

### 2.6. Summary Measures and Data Synthesis

Prior to any decision to quantitatively pool together data from individual studies, clinical heterogeneity was examined in terms of individual study settings and conditions, population characteristics, eligibility criteria, or analyses. Statistical heterogeneity was examined, first visually, through inspection of the confidence bounds within the forest plots, as well as statistically, as indicated by a *p*-value below the level of 10% for the test (*p* < 0.10). An I^2^ test for homogeneity was also undertaken to quantify the extent of heterogeneity.

Random effects meta-analyses were conducted as they are considered more appropriate to incorporate individual study findings. In view of the anticipated dichotomous nature of the expected outcomes, pooled treatment effects were calculated through risk ratios (RRs) with associated 95% confidence intervals (95% CIs) and prediction intervals (95% PIs). Study authors were contacted when additional data and information were required that could not be retrieved by the publication record.

### 2.7. Risk of Bias across Studies

Publication bias was explored through standard funnel plots and Egger’s regression test [24].

### 2.8. Additional Analyses

Sensitivity analyses were considered, if applicable, to explore and isolate the effect of studies with serious/critical risk of bias on the overall effect, if studies of both serious/critical or lower risk of bias were included. In addition, the effect of studies including participants with well-known underlying diseases (i.e., diabetes) in the final sample, or smoking, was removed by excluding the studies from separate sensitivity analyses. If a wide age range was to be identified across included studies, subgroup analyses were also considered as appropriate. Recording of outcomes based on patient self-reporting was also considered separately through sensitivity analysis. Lastly, an all-bias inclusive sensitivity analysis was performed by excluding all aforementioned potential sources of uncontrolled confounding or bias.

### 2.9. Assessment of the Quality of the Evidence

The Grading of Recommendations Assessment, Development and Evaluation (GRADE) was implemented to assess the overall quality of the evidence as formulated by the conditions/exposures and outcomes for evaluation. According to GRADE, the overall body of evidence is rated as high, moderate, low, and very low. The ratings, with regard to the likelihood for a change in our confidence in the estimated effect, range from very unlikely to very likely, to be modified [25,26]. Assessment of the body of evidence primarily involves assessment of study design. Assessment is made on the following domains: risk of bias, inconsistency, indirectness, imprecision, and publication bias. For the first four domains, the quality of evidence may be downgraded on the basis of either ‘serious’ or ‘very serious’ risks (1 or 2 levels, respectively); publication bias may either be suspected or undetected. For non-randomized/observational designs specifically, which theoretically start from a ‘low’ level of evidence, the perspectives for upgrade are as follows: a large or very large effect, a plausible residual confounding that may alter the effect, or a dose–response gradient. The level of evidence may be upgraded by 1 or 2 levels (large effect), or 1 level (plausible confounding, dose–response gradient).

## 3. Results

### 3.1. Search Details

**Figure 1:**
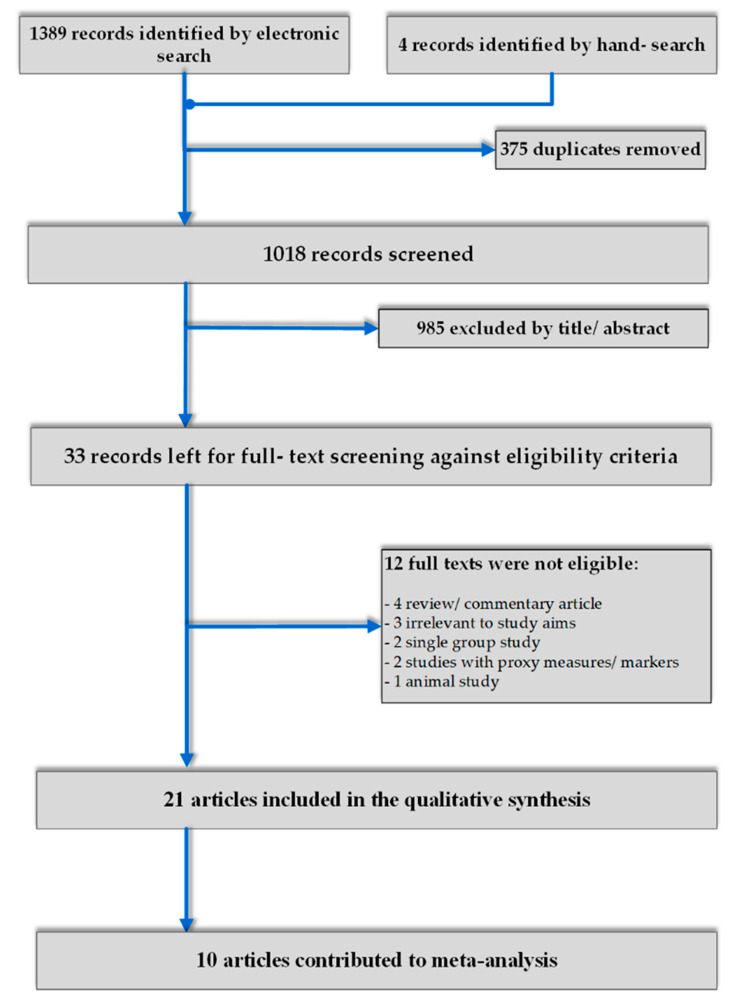
Flow chart of study selection.

The study selection process and the ultimate number of included articles in qualitative and quantitative synthesis is presented in Figure 1. From an initial hit of 1393 results, 21 articles passed through a full-text screening process to the qualitative synthesis [14,15,16,18,19,27,28,29,30,31,32,33,34,35,36,37,38,39,40,41,42]. Of those, 10 papers contributed to meta-analyses and/or additional sensitivity analysis [14,15,18,27,28,31,36,37,40,41]. Reasons for exclusion of articles are outlined in Figure 1.

### 3.2. Study Design and Characteristics

Detailed characteristics of included studies are presented in Table 1. Of the 21 studies, six cohort studies were identified—four retrospective and two prospective—while the majority (n = 14) were of cross-sectional design. Only one case-control study was detected. Sample sizes for cohort studies ranged from 278 [31] to 283,590 [35]. The respective numbers for cross-sectional studies were 55 [30] to 666,768 [18] participants, while the sole case-control study reported 100 patients [36]. Three studies involved only male patients [14,16,33], one study only female patients [29], while the rest involved both sexes. The reported age of participants was more than a mean of 45 years for the two-thirds of the included studies (n = 14), while seven studies included younger age ranges in conjunction with older or in isolation [14,16,18,28,30,32,35].

Cardiovascular disease entities and cases identified as outcomes in the present study involved mostly hard measures of prevalence/incidence of CVDs in general, coronary heart disease, including coronary artery disease, coronary artery atherosclerosis/atherosclerotic burden, myocardial infraction, or unstable angina. Only a small proportion of studies involved other proxy measures for CVD outcomes, such as C-reactive protein, flow-mediated dilation, or hypertension [16,30,38,39]. Chronic endodontic infection was vastly confirmed through radiographic examination in all but one study [19]. The diagnosis of the endodontic condition described by most studies pertained to the reporting of diagnosis of “apical periodontitis”, “lesion of endodontic origin”, “endodontic pathology”, and “periapical destruction”. In a small proportion of studies, the description of the condition pertained to endodontic therapy, root canal treatment, and unfinished root canal treatment [19,33,35]. The latter do not constitute robust and definite measures of infections of endodontic origin, or even proxies for this, within the frame of the designs under investigation, and as such, they were not considered for the quantitative synthesis (Table 1).

### 3.3. Risk of Bias within Studies

Overall, risk of bias was rated as moderate in 11 of the included studies, while it was rated as serious in the remaining 10. The most severely impacted domains were confounding by potentially undisclosed, undetermined, or non-controlled risk factors, classification of the conditions/exposures, and measurement of the outcomes. Both latter domains pertain to a potential lack of blinding of the investigators, who were involved in the assessment of either the exposure or the outcome. In addition, although the specific domain of “selection of the reported result” was uniformly classified as “low risk of bias”, it should be noted that none of the included studies described pre-registration of a related protocol for the study; however, correspondence between the described methodology in the article and reported analyses and results was considered acceptable (Table 2; Table A1).

### 3.4. Effects of Interventions, Meta-Analyses, Additional Analyses

**Figure 2:**
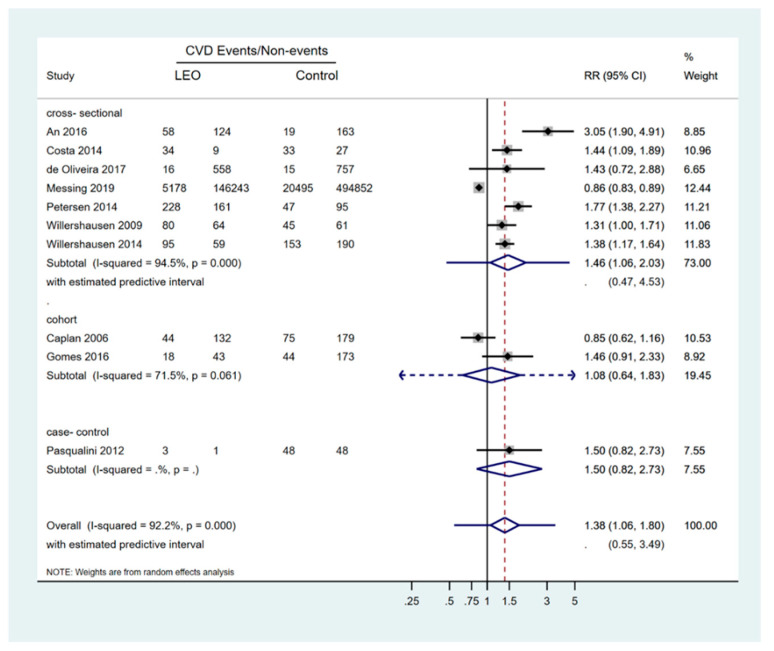
Forest plot for random effects meta-analyses for the overall effect of LEO (lesions of endodontic origin) and CVD (cardiovascular disease) events.

As previously noted, 10 studies contributed to meta-analysis or additional analyses [14,15,18,27,28,31,36,37,40,41]. Studies reporting on any hard cardiovascular outcomes as described above, excluding proxy measures, and definite documentation of lesions of endodontic origin/apical periodontitis were considered eligible for quantitative synthesis. According to the overall estimate for the perceived association between the two entities (i.e., lesion of endodontic origin and CVDs), patients with a diagnosis of chronic endodontic infection/apical periodontitis had 38% higher risk of being diagnosed with a CVD (10 studies: RR = 1.38; 95%CI: 1.06, 1.80; *p* = 0.008); however, prediction intervals (95%PIs) included the null and were recorded as 0.55 to 3.49, illustrating the variability of the true effect in different conditions, studies, or settings. Furthermore, although the effect was not obvious solely for the two cohort studies eligible for inclusion in the analysis (two studies, subgroup analysis: RR = 1.08; 95%CIs: 0.64, 1.83; *p* = 0.78), the results of the meta-regression analysis, with Knapp–Hartung modification, built to identify any effect of the study design on the summary effect, did not reveal a significant association (*p* = 0.61). In essence, the scarcity of studies of this design included in the meta-analyses might have blurred the real effect, providing a decreased power to identify the association (Table 3, Figure 2).

Sensitivity analysis considered age range, baseline similarity of important confounders (matching across groups on diabetes/smoking), and self-reporting of outcomes assessed, and the results of these analyses are outlined in Table 3. Evidently, the effect pertained to all subsequent analyses: for example, when only patients more than a mean of 45 years of age were included, the associated risk for CVDs elevated to 46% higher for patients with chronic endodontic infection/apical periodontitis (RR = 1.46; 95%CIs: 1.19, 1.79; *p* < 0.001), as opposed to those without. Likewise, when only studies with moderate risk of bias were included [15,31,36,37], the effect appeared stronger, with RR = 1.86 (95%CIs: 1.37, 2.52; *p* < 0.001) and apparently lower heterogeneity across the subsample (I^2^ = 50.7; *p* = 0.11).

**Figure 3:**
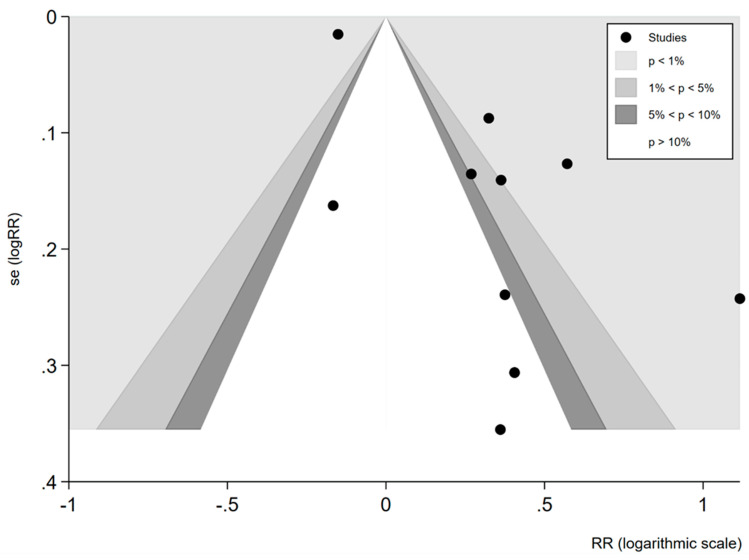
Contour-enhanced funnel plot for inspection of publication bias.

Publication bias was detected. A contour-enhanced funnel plot revealed a relative absence of published studies with null effect and non-significant results. Studies were missing from the inner central and lower left part of the plot (Figure 3), while Egger’s test for small-study effects was apparently significant (*p* = 0.003).

### 3.5. Quality of the Evidence

The quality of the existing evidence for the association between chronic endodontic infection/apical periodontitis and CVDs was very low. The design of the included studies along with decisions to downgrade the quality of the evidence due to between-study heterogeneity and publication bias issues contributed to this rating (Table A2). Apparently, this means that we are uncertain about the effect estimate, and further research is very likely to change our confidence with regard to this effect.

## 4. Discussion

### 4.1. Findings in Context

A rough estimator of the results of the present review has indicated great uncertainty with regard to the perceived association between endodontic infections and CVDs. In essence, there appears to be a substantial amount of individual attempts to elucidate the causal mechanisms of these associations; however, most research endeavors have failed to prove successful, as study design and potential confounders or independently but simultaneously co-acting factors continue to impose a non-negligible amount of obscurity on causality pathways. Thus, the identification of an elevated risk for cardiovascular-related diseases, as implied by the present synthesis of available evidence, should be placed into the appropriate context, conditional on the retrieved weaknesses of the included original study designs.

Effectively, efforts have been ongoing for the last 15 years, and with an increasing intensity in the past 5 to 6 years, to shed light on regional, endodontic infections and their impact on general health either in the short or long term. This observation might have been partially endorsed by the most recent estimations for an anticipated increase in the prevalence of CVDs within the years to come of approximately 10% [43]; moreover, it is also endorsed by the WHO estimations for approximately 24 million patients losing their lives to CVDs by the year 2030 [3]. Interestingly, lately, CVDs have been associated with the path and prognosis of COVID-19 patients [44], with autopsy material confirming a cytokine-mediated exacerbation and related inflammation in CVD patients also positive for COVID-19, thus pinpointing the central role of a call for further endorsements on the investigation and research against any potential risk factor for CVDs, as seasonal epidemics or pandemics may potentially pose a greater burden for the morbidity of the disease at present or in the near future.

Cardiovascular entities entail a large group of non-communicable diseases framed under coronary heart disease, stroke, atherosclerosis, and myocardial infraction and affecting the human heart and blood vessels [45]. A key and commonly shared element is the presence of vascular events and systemic implications for the individual. To this end, it has been suggested that various factors might be implicated in the pathogenic mechanisms of such a condition, including underlying grounds of diabetes mellitus and rheumatoid arthritis [46]. An important consideration when evaluating the onset of CVDs is the close investigation of other entities that may run ahead and parallel with the CVDs and are effectively served by inflammation, tissue damage, and macrovascular circulation-related issues [47,48]. The results include the development of atheromatosis or atherosclerosis, carotid plaque, and luminal narrowing of arteries [49], with most severe endpoints being stroke, myocardial infraction, and mortality. A non-negligible amount of studies included in the present review involved participants with underlying diabetes disease, thus potentially serving as a positive confounder for the investigated association; however, sensitivity analysis, after the elimination of those studies, indicated the persistence of the recorded effect and its direction.

### 4.2. Prior Research

In the same line and following the scheme for identifying common pathogenic mechanisms and/or triggering factors for the development of a potentially disrupting environment for the heart and vessels, researchers in dental medicine have been studying the effect of chronic infections of endodontic origin (i.e., pulp necrosis/root canal infection) on CVDs [14,15,18].

Acute inflammation of dental pulp, due to caries or traumatic injury, may result in pulp necrosis and subsequent tissue infection, bearing an increased dynamic to induce the formation of apical periodontitis in proximity to the infected internal tooth structures [50]. A failed root canal treatment may also serve as a proxy for the initiation of apical periodontitis. Such lesions of endodontic origin appear as a result of bacterial and microbial pathogen transmission from the infected pulp space to the periapical region of the tooth; in essence, bone destruction in the periapical region is additionally described as the endpoint of a surplus immune system reaction to inflammation [51]. A range of inflammatory biomarkers have been recognized as playing a central role in the pathogenesis and physiology of lesions of endodontic origin. A very recent review has outlined the basis of inflammatory response to apical periodontitis and has elucidated the biologic mechanisms involved. Integral compounds of this are neutrophils at the initial and most acute phase followed by the recruitment and infiltration of mast cells and macrophages as a second-line response, which are allied to the chronicity of infection. What is more, prostaglandins, cytokines, and chemokines produced are associated with the recruitment of leukocytes [51,52,53].

Evidently, potential associations with the advent of CVDs might be presumed and considered biologically plausible, since bacteria that originated from infected tissues coupled with by-products of the inflammatory process and the formation of granulomas might interact and enter systemic circulation with an impact on major blood vessels. Interestingly, efforts to ameliorate the progression of chronic apical periodontitis have been reported to present a positive effect on endothelial dysfunction biomarkers, as demonstrated by a successful root canal treatment of the affected teeth [54]. A similar picture has long been elucidated for periodontal disease and cardiovascular outcomes [8], while lately, research has been targeted towards the discovery of shared genetic background and risk loci of the two entities [55].

### 4.3. Clinical Implications

Despite weak evidence for an association between chronic endodontic infection/apical periodontitis and CVDs, additional to the limitations of the included studies, clinical cardiologists should consider chronic endodontic infections as potential proxies for adverse effects in patients with CVDs. A similar association has already been acknowledged for other oral pathologic conditions such as periodontal disease [8]. To this end, clinical and radiographic evaluation of patients’ oral condition, teeth, and supporting tissues is paramount. Dentists and oral health practitioners may play a central role in prevention strategies related to prognostic factors for CVDs. Moreover, the radiographic examination of patients with CVDs through standard panoramic radiographs may aid to accurately detect existing periapical inflammatory lesions and advise patients to proceed to further clinical examination to assess whether these lesions are active or not, thus minimizing any potential risk for systemic effects of inflammatory mediators and bacteremia.

### 4.4. Strengths and Limitations

Compared to prior reports in the field [56,57,58,59], this is the first systematic attempt that comprehensively and transparently assessed all available scientific evidence, with a quantitative synthesis of 10 studies, offering increased precision regarding the estimated effect and association, while focusing on placing available evidence from potentially variant sources under the appropriate context in terms of research quality, quality of the evidence, and/or reporting quality. The presence of significant shortcomings of prior studies in the field, such as heterogeneity issues related to patients’ characteristics and studies’ settings, or confounding effects were also addressed with appropriate sensitivity analyses, which added to the methodological robustness of the present report. In addition, only hard cardiovascular outcomes were considered in the meta-analyses, excluding biomarkers or proxy measures that might have suffered from uncertainty in the establishment of a certain diagnosis. After pre-registration to an openly available framework [20], a systematic search was conducted in published and unpublished literature across seven databases, while this is to our knowledge the first formal attempt to statistically and visually explore the presence of publication bias in the field; this held true, based on the estimated effects of the retrieved studies alongside their variances. Furthermore, the quality of the existing evidence and its implications were assessed and explored with the GRADE framework, rendering initiatives for further well-conducted and reported research highly anticipated.

The study was not free of limitations, though. First, the internal validity of the included studies was in broad terms suboptimal, thus impacting on the interpretation of the results of the conducted syntheses. Second, the majority of studies were of cross-sectional design, with additional bias-related issues--for example, self-reporting of endodontic lesions—or inclusion of other co-morbidities in the samples achieved and non-confirmed temporal sequence of the investigated conditions. In addition, the possibility of the long-term silent existence of a necrotic pulp only, without periradicular spread of the infection or inflammation and thus apparently forming a reduced probability of detection through radiographic examinations, might be an additional factor related to underestimation of the perceived association; however, this is always conditional on the study design.

## 5. Conclusions

In consideration of all identified and discussed caveats, an association between chronic endodontic infection and CVDs may not be disregarded, although being of limited evidence quality at present, because the biologic background of such a relation is plausible. Targeting and identifying any single risk factor for a worldwide health-impacting disease seems imperative, which must be based on the most high-quality evidence. Certainly, further research with additional focus on the temporal circumstances and establishment of the association under study, while also on vastly well-conducted and reported studies, should be greatly valued. 

## Figures and Tables

**Table 1:** Characteristics of included studies (n = 21).

Study ID/Design	Population	Exposure	Outcome	Notes
**An et al., 2016** Cross-sectional (pair-matched)	362 patients (266 female, 98 male); mean age 49	AP (+/−), radiographic diagnosis	Prevalence of CVD, HCA	Other underlying disease in part of the sample: diabetes
**Caplan et al., 2006** Cohort (prospective)	708 patients (all male); age split to <45 yrs or >45 yrs	LEO (+/−), radiographic diagnosis	Time to diagnosis of CHD	Longitudinal cohort, splits patients < or > 45 yrs; other underlying disease in part of the sample: diabetes
**Chauhan et al., 2019** Cross-sectional (pair-matched)	120 patients (all male); 20–40 yrs	AP (+/−), radiographic diagnosis	FMD, c-IMT	Physiologic and anatomic measures of early CVD
**Costa et al., 2014** Cross-sectional	103 patients (52 male, 51 female); mean age 61.9 yrs	AP (+/−), radiographic diagnosis	Prevalence of CAD	Other underlying disease in part of the sample: diabetes
**Cowan et al., 2020** Cohort (population-based prospective)	6274 patients (based on CHD data) (2966 male, 3308 female); mean age 62.3 ± 5.7, for non-ET/62.7 ± 5.7 for ET cases	Self-reported history of ET	Incidence of CHD	Other underlying disease in part of the sample: diabetes, periodontal disease; ET as proxy of infection and also self-reported
**deOliviera et al., 2017** Cross-sectional	1346 patients (438 male, 908 female); wide age range: ≤18 and 19 to ≥60 yrs	AP (+/−), radiographic diagnosis	Prevalence of CAD	
**Friedlander et al., 2010** Cross-sectional (pair-matched)	72 patients (70 male, 72 female); cases mean age: 64.4 ± 10.0; controls mean age: 64.9 ± 10.1	CAA (+/−)	CDI as measured by MPI	MPI noted as the outcome, after matching on CAA presence
**Frisk et al., 2003** Cross-sectional	1056 patients (all female); mean age: 64.7 ± 10.7	PA (+/−), radiographic diagnosis	Prevalence of CVD	Other underlying disease in part of the sample: diabetes
**Garrido et al., 2019** Cross-sectional	55 patients (32 male, 23 female); cases mean age: 25.9 ± 5.0; controls mean age: 24.5 ± 3.9	LEO (+/−), radiographic diagnosis	CVD, (hsCRP)	Blood samples for other proxy markers for CVD risk as well
**Gomes et al., 2016** Cohort (retrospective)	278 patients (143 male, 135 female); age: 55 ± 16.8 yrs	AP (+/−), radiographic diagnosis	Incidence of CHD	Other underlying disease in part of the sample: diabetes
**Jansson et al., 2001** Cohort (retrospective)	1393 patients (687 male, 706 female); age range: 18–66 yrs	AP (+/−), radiographic diagnosis	Prevalence of CVD (mortality)	
**Joshipura et al., 2006** Cohort (retrospective)	34,683 patients (male); age range: cases mean age: 54.2 ± 9.1; controls mean age: 52.1 ± 9.2	Pulpal inflammation leading to RCT (+/−)	Incidence of CHD	Self-reported root canal for inflammation as surrogate; sample consisted of health professionals
**Liljestrand et al., 2016** Cross-sectional	508 patients (330 male, 178 female); age: 62.1 (±10.4)	Prevalence CAD, ACS	LEO (+/−), radiographic diagnosis	Other underlying disease in part of the sample: diabetes; no clear breakdown of LEOs
**Lin et al., 2015** Cohort (population-based retrospective)	283,590 participants (123804 male, 159786 female); age range: 20- >60	Unfinished RCT (+/−)	First diagnosis of CVD hospitalization	Other underlying disease in part of the sample: diabetes
**Messing et al., 2019** Cross-sectional	666.768 patients; age range: 18–65	EP (+/−),	Prevalence all CVDs combined (self-reported)	Epidemiologic association plus genetic association study
**Pasqualini et al., 2012** Case-control	100 patients; cases age: 48 ± 5.7; controls age: 47 ± 7.1	Acute myocardial infraction/unstable angina	LEO (+/−), clinical, and radiographic	Identification of polymorphisms CD14
**Petersen et al., 2014** Cross-sectional	531 patients (1191 teeth); age 50 ± 15.7	AP (+/−), radiographic diagnosis CT scan	Volume of aortic atherosclerotic burden	Large number of subgroups
**Segura-Egea et al., 2010** Cross-sectional	91 patients (21.2 teeth/patient); cases age: 59.5 ± 9.7; controls age: 56.4 ± 9.9	Hypertension (+/−)	AP (+/−), radiographic diagnosis	Underlying diseases (diabetes, smoke) not recorded in the sample
**Virtanen et al., 2017** Cross-sectional	120 patients (57 male, 63 female); cases age: 53.0 ± 2.7; controls age: 51.4 ± 2.9	AP (+), radiographic diagnosis	Prevalence of CVD	Most CVD cases were classified as hypertensive (no specific number provided)
**Willershausen et al., 2009** Cross-sectional	250 patients (203 male, 47 female); cases age: 61.8 ± 10.4; controls age: 63.4 ± 10.7	Myocardial infraction (+/−)	Prevalence of LEO (+/−)	Other underlying disease in part of the sample: diabetes
**Willershausen et al., 2014** Cross-sectional	497 patients; mean age 62.3, range 51–83	Acute myocardial infraction (+/−)	Prevalence of LEO (+/−)	Lesions of periodontal origin also recorded

ACS, acute coronary syndrome; AP, apical periodontitis; CAA, coronary artery atherosclerosis; CAD, coronary artery disease; CHD, coronary heart disease; CDI, chronic dental infection; c-IMT, intima-media thickness; CT, computed tomography; CVD, cardiovascular disease; EP, endodontic pathology; ET, endodontic treatment; FMD, flow-mediated dilatation; HCA hypercholesterolemia; hsCRP, high-sensitivity C-reactive protein; LEO, lesions of endodontic origin; MPI, Mattila pantomography index; PA, periapical destruction; yrs, years; RCT, root canal treatment.

**Table 2:** Risk of bias according to the ROBINS-I tool for the included studies (n = 21).

	Bias Due to/in…							
	Confounding	Selection of Participants into the Study	Classification of Interventions	Deviations from Intended Interventions	Missing Data	Measurement of Outcomes	Selection of the Reported Result	Overall
An et al., 2016	**Moderate**	**No Information**	**Low**	**Low**	**Low**	**Low**	**Low**	**Moderate**
Caplan et al., 2006	**Low**	**No Information**	**Low**	**Low**	**Serious**	**Low**	**Low**	**Serious**
Chauhan et al., 2019	**Serious**	**No Information**	**Low**	**Low**	**Low**	**Low**	**Low**	**Serious**
Costa et al., 2014	**Moderate**	**No Information**	**Serious**	**Low**	**Low**	**Moderate**	**Low**	**Serious**
Cowan et al., 2020	**Low**	**Low**	**Low**	**Low**	**Low**	**Moderate**	**Low**	**Moderate**
deOliviera et al., 2017	**Serious**	**No Information**	**Serious**	**Low**	**Low**	**Serious**	**Low**	**Serious**
Friedlander et al., 2010	**Serious**	**No Information**	**Low**	**Low**	**Low**	**Low**	**Low**	**Serious**
Frisk et al., 2003	**Moderate**	**No Information**	**Serious**	**Low**	**Low**	**Serious**	**Low**	**Serious**
Garrido et al., 2019	**Moderate**	**No Information**	**Serious**	**Low**	**Low**	**Serious**	**Low**	**Serious**
Gomes et al., 2016	**Moderate**	**No Information**	**Low**	**Low**	**Low**	**Low**	**Low**	**Moderate**
Jansson et al., 2001	**Moderate**	**No Information**	**Low**	**Low**	**Low**	**Moderate**	**Low**	**Moderate**
Joshipura et al., 2006	**Moderate**	**No Information**	**Low**	**Low**	**Low**	**Low**	**Low**	**Moderate**
Liljestrand et al., 2016	**Moderate**	**No Information**	**Low**	**Low**	**Low**	**Low**	**Low**	**Moderate**
Lin et al., 2015	**Moderate**	**No Information**	**Low**	**Low**	**Low**	**Moderate**	**Low**	**Moderate**
Messing et al., 2019	**Serious**	**No Information**	**Low**	**Low**	**Low**	**Moderate**	**Low**	**Serious**
Pasqualini et al., 2012	**Moderate**	**No Information**	**Low**	**Low**	**Low**	**Low**	**Low**	**Moderate**
Petersen et al., 2014	**Moderate**	**No Information**	**Low**	**Low**	**Low**	**Moderate**	**Low**	**Moderate**
Segura-Egea et al., 2010	**Moderate**	**No Information**	**Serious**	**Low**	**Low**	**Moderate**	**Low**	**Serious**
Virtanen et al., 2017	**Moderate**	**No Information**	**Low**	**Low**	**Moderate**	**Low**	**Low**	**Moderate**
Willershausen et al., 2009	**Moderate**	**No Information**	**Serious**	**Low**	**Low**	**Moderate**	**Low**	**Serious**
Willershausen et al., 2014	**Moderate**	**No Information**	**Serious**	**Low**	**Low**	**Moderate**	**Low**	**Serious**

**Table 3:** Results of meta-analyses, sensitivity analyses.

Synthesis	No. Studies	Risk Ratio	95%CIs	*p*-Value	I^2^ (%)	Tau-Squared
**Overall**	**10**	**1.38**	**1.06, 1.80**	**<0.001**	**92.2**	**0.13**
Age ^1^	8	1.46	1.19, 1.79	<0.001	70.6	0.05
Matching ^2^	8	1.36	1.02, 1.82	0.04	93.5	0.15
Self-reporting ^3^	9	1.46	1.21, 1.77	<0.001	66.4	0.05
All-sensitivity ^4^	6	1.46	1.15, 1.86	0.002	79.0	0.07
Bias ^5^	4	1.86	1.37, 2.52	<0.001	50.7	0.05

^1^ two studies with a wide age range (including patients less than 45 years of age) have been excluded. ^2^ two studies with unequal distribution of important potential confounders (i.e., diabetes, smoking) across sample groups have been excluded. ^3^ one study has been excluded due to assessment based on patient self-reporting for the cardiovascular outcome. ^4^ four studies have been excluded for all the above reasons cumulatively. ^5^ four studies with moderate risk of bias were included; the remaining six with serious risk of bias have been excluded.

## Data Availability

Data are available by the corresponding author upon reasonable request.

## Appendix A

MEDLINE via Pubmed. Date of search: 2.10.2020. Limits: none. Hits: 863.

(apical periodontitis) OR (chronic apical periodontitis) OR (apic * periodont *) OR (tooth periapical lesion) OR (teeth periapical lesion) OR (tooth apical lesion) OR (teeth apical lesion) OR (chronic tooth inflammation) OR (chronic pulp teeth inflammation) OR (chronic teeth inflammation) OR (radiolucent periapical teeth lesion) OR (radiolucent periapical tooth lesion) OR (tooth root canal treatment) OR (endodontic treatment) OR (root canal tooth therapy) OR (endodontic infection) OR (tooth pulp* infection) OR (chronic dental infection)) AND (coronary disease) OR (coronary heart disease) OR (cardiovascular disease) OR (hypertension) OR (atherosclerosis) OR (CVD) OR (stroke) OR (myocardial infraction)) NOT (diabetes) OR (metabolic)).

Table A1 Detailed assessment of ROBINS-I tool for the included studies (supplement to Table 2).

**Table:** (**A**)

Domain	Reference	An et al., 2016	Caplan et al., 2006	Chauhan et al., 2019	Costa et al., 2014	Cowan et al., 2020	De Oliveira et al., 2017	Freidlander et al., 2010	Frisk et al., 2003	Garrido et al., 2019
1. Confounding	1.1	PY	PN	PY	PY	PN	PY	PY	PY	PY
	1.2	NA	NA	NA	NA	NA	NA	NA	NA	NA
	1.3	NA	NA	NA	NA	NA	NA	NA	NA	NA
	1.4	PY	NA	PN	PY	NA	PN	PN	PY	PY
	1.5	PY	NA	NA	PY	NA	NA	NA	PY	PY
	1.6	NA	NA	NI	NA	NA	NI	NI	NA	NA
	1.7	NA	NA	PN	NA	NA	PN	PN	NA	NA
	1.8	NA	NA	NA	NA	NA	NA	NA	NA	NA
	Judgement	Moderate	Low	Serious	Moderate	Low	Serious	Serious	Moderate	Moderate
2. Selection of participants into the study	2.1	N	N	PN	PN	PN	PN	PN	PN	PN
	2.2	NA	NA	NA	NA	NA	NA	NA	NA	NA
	2.3	NA	NA	NA	NA	NA	NA	NA	NA	NA
	2.4	NI	NI	NI	NI	NI	NI	NI	NI	NI
	2.5	NA	NA	NA	NA	NA	NA	NA	NA	NA
	Judgement	NI	NI	NI	NI	NI	NI	NI	NI	NI
3. Classification of interventions	3.1	PY	Y	PY	PY	Y	PY	Y	PY	PY
	3.2	PY	Y	PY	PY	Y	PY	Y	PY	PY
	3.3	N	N	N	PY	N	PY	N	PY	PY
	Judgement	Low	Low	Low	Serious	Low	Serious	Low	Serious	Serious
4. Deviations from intended interventions	4.1	PN	PN	PN	PN	PN	PN	PN	PN	PN
	4.2	NA	NA	NA	NA	NA	NA	NA	NA	NA
	4.3	NA	NA	NA	NA	NA	NA	NA	NA	NA
	4.4	NA	NA	NA	NA	NA	NA	NA	NA	NA
	4.5	NA	NA	NA	NA	NA	NA	NA	NA	NA
	4.6	NA	NA	NA	NA	NA	NA	NA	NA	NA
	Judgement	Low	Low	Low	Low	Low	Low	Low	Low	Low
5. Missing data	5.1	Y	PN	Y	Y	PY	PY	Y	PY	Y
	5.2	N	PN	N	N	N	PN	N	PN	N
	5.3	N	PY	N	N	PN	PN	N	PN	N
	5.4	NA	NI	NA	NA	NA	NA	NA	NA	NA
	5.5	NA	NI	NA	NA	NA	NA	NA	NA	NA
	Judgement	Low	Serious	Low	Low	Low	Low	Low	Low	Low
6. Measurement of outcomes	6.1	PN	PN	PN	PY	PY	PY	PN	PY	PY
	6.2	N	N	N	NI	NI	NI	N	NI	NI
	6.3	PY	PY	PY	PY	PY	NI	PY	NI	NI
	6.4	PN	PN	PN	PN	PN	NI	PN	NI	NI
	Judgement	Low	Low	Low	Moderate	Moderate	Serious	Low	Serious	Serious
7. Selection of the reported result	7.1	PN	PN	PN	PN	PN	PN	PN	PN	PN
	7.2	PN	PN	PN	PN	PN	PN	PN	PN	PN
	7.3	PN	PN	PN	PN	PN	PN	PN	PN	PN
	Judgement	Low	Low	Low	Low	Low	Low	Low	Low	Low
Overall	Judgement	Moderate	Serious	Serious	Serious	Moderate	Serious	Serious	Serious	Serious

Y, yes; PY, probably yes; N, no; PN, probably no; NI, no information; NA, not applicable.

**Table:** (**B**)

Domain	Reference	Gomes et al., 2016	Jansson et al., 2001	Joshipura et al., 2006	Liljestrand et al., 2016	Lin et al., 2015	Messing et al., 2019	Pasqualini et al., 2012	Petersen et al., 2014
1. Confounding	1.1	PY	PY	PY	PY	PY	PY	PY	PY
	1.2	NA	NA	NA	NA	NA	NA	NA	NA
	1.3	NA	NA	NA	NA	NA	NA	NA	NA
	1.4	PY	PY	PY	PY	PY	PN	PY	PY
	1.5	PY	PY	PY	PY	PY	PN	PY	PY
	1.6	NA	NI	NI	NA	NA	NA	NA	NA
	1.7	NA	NA	NA	NA	NA	NA	NA	NA
	1.8	NA	NA	NA	NA	NA	NA	NA	NA
	Judgement	Moderate	Moderate	Moderate	Moderate	Moderate	Serious	Moderate	Moderate
2. Selection of participants into the study	2.1	N	PN	PN	PN	N	PN	PN	PN
	2.2	NA	NA	NA	NA	NA	NA	NA	NA
	2.3	NA	NA	NA	NA	NA	NA	NA	NA
	2.4	NI	NI	NI	NI	NI	NI	NI	NI
	2.5	NA	NA	NA	NA	NA	NA	NA	NA
	Judgement	NI	NI	NI	NI	NI	NI	NI	NI
3. Classification of interventions	3.1	PY	PY	PY	PY	PY	PY	PY	PY
	3.2	PY	PY	PY	PY	PY	PY	PY	PY
	3.3	N	PN	PN	PN	PN	PN	N	PN
	Judgement	Low	Low	Low	Low	Low	Low	Low	Low
4. Deviations from intended interventions	4.1	PN	PN	PN	PN	PN	PN	PN	PN
	4.2	NA	NA	NA	NA	NA	NA	NA	NA
	4.3	NA	NA	NA	NA	NA	NA	NA	NA
	4.4	NA	NA	NA	NA	NA	NA	NA	NA
	4.5	NA	NA	NA	NA	NA	NA	NA	NA
	4.6	NA	NA	NA	NA	NA	NA	NA	NA
	Judgement	Low	Low	Low	Low	Low	Low	Low	Low
5. Missing data	5.1	Y	PY	PY	PY	Y	PY	PY	Y
	5.2	N	PN	PN	PN	PN	PN	PN	N
	5.3	N	PN	PN	PN	PN	PN	PN	N
	5.4	NA	NA	NA	NA	NA	NA	NA	NA
	5.5	NA	NA	NA	NA	NA	NA	NA	NA
	Judgement	Low	Low	Low	Low	Low	Low	Low	Low
6. Measurement of outcomes	6.1	PN	PN	PN	PN	PN	PN	PN	PY
	6.2	N	NI	N	N	NI	NI	N	NI
	6.3	PY	PY	PY	PY	PY	PY	PY	PY
	6.4	PN	PN	PN	PN	PN	PN	PN	PN
	Judgement	Low	Moderate	Low	Low	Moderate	Moderate	Low	Moderate
7. Selection of the reported result	7.1	PN	PN	PN	PN	PN	PN	PN	PN
	7.2	PN	PN	PN	PN	PN	PN	PN	PN
	7.3	PN	PN	PN	PN	PN	PN	PN	PN
	Judgement	Low	Low	Low	Low	Low	Low	Low	Low
Overall	Judgement	Moderate	Moderate	Moderate	Moderate	Moderate	Serious	Moderate	Moderate

Y, yes; PY, probably yes; N, no; PN, probably no; NI, no information; NA, not applicable.

**Table:** (**C**)

Domain	Reference	Segura-Egea et al., 2010	Virtanen et al., 2017	Willershausen et al., 2009	Willershausen et al., 2014
1. Confounding	1.1	PY	PY	PY	PY
	1.2	NA	NA	NA	NA
	1.3	NA	NA	NA	NA
	1.4	PY	PY	PY	PY
	1.5	PY	PY	PY	PY
	1.6	NA	NA	NA	NA
	1.7	NA	NA	NA	NA
	1.8	NA	NA	NA	NA
	Judgement	Moderate	Moderate	Moderate	Moderate
2. Selection of participants into the study	2.1	N	PN	PN	PN
	2.2	NA	NA	NA	NA
	2.3	NA	NA	NA	NA
	2.4	NI	NI	NI	NI
	2.5	NA	NA	NA	NA
	Judgement	NI	NI	NI	NI
3. Classification of interventions	3.1	PY	PY	PY	PY
	3.2	PY	PY	PY	PY
	3.3	PY	N	PY	PY
	Judgement	Serious	Low	Serious	Serious
4. Deviations from intended interventions	4.1	PN	PN	PN	PN
	4.2	NA	NA	NA	NA
	4.3	NA	NA	NA	NA
	4.4	NA	NA	NA	NA
	4.5	NA	NA	NA	NA
	4.6	NA	NA	NA	NA
	Judgement	Low	Low	Low	Low
5. Missing data	5.1	Y	PN	Y	PY
	5.2	N	PN	N	PN
	5.3	N	PN	N	PN
	5.4	NA	NI	NA	NA
	5.5	NA	NI	NA	NA
	Judgement	Low	Moderate	Low	Low
6. Measurement of outcomes	6.1	PY	PN	PY	PY
	6.2	NI	N	NI	NI
	6.3	PY	PY	PY	PY
	6.4	PN	PN	PN	PN
	Judgement	Moderate	Low	Moderate	Moderate
7. Selection of the reported result	7.1	PN	PN	PN	PN
	7.2	PN	PN	PN	PN
	7.3	PN	PN	PN	PN
	Judgement	Low	Low	Low	Low
Overall	Judgement	Serious	Moderate	Serious	Serious

Y, yes; PY, probably yes; N, no; PN, probably no; NI, no information; NA, not applicable.

**Table A2:** Summary of Findings Table/GRADE assessment.

Association Between Chronic Endodontic Infection and Cardiovascular Disease						
Patient or population: any Settings: population studies Exposure: chronic dental infection Outcome: cardiovascular event						
**Outcomes**	**Illustrative comparative risks * (95% CI)**		**Relative effect** **(95% CI)**	**No of** **participants** **(studies)**	**Quality of the** **evidence** **(GRADE)**	**Comments**
	Assumed risk	Corresponding risk				
**Cardiovascular Event**	**41 per 1000**	**56 per 1000** (43 to 73)	**RR 1.38** (1.06 to 1.80)	670,668 (10 studies)	⊕⊝⊝⊝ **very low** ^1,2^	-

* The basis for the **assumed risk** (e.g., the median control group risk across studies) is provided in footnotes. The **corresponding risk** (and its 95% confidence interval) is based on the assumed risk in the comparison group and the **relative effect** of the exposure (and its 95% CI). **CI:** confidence interval; **RR:** risk ratio; GRADE Working Group grades of evidence. **High quality:** Further research is very unlikely to change our confidence in the estimate of effect. **Moderate quality:** Further research is likely to have an important impact on our confidence in the estimate of effect and may change the estimate. **Low quality:** Further research is very likely to have an important impact on our confidence in the estimate of effect and is likely to change the estimate. **Very low quality:** We are very uncertain about the estimate; ^1^ downgraded once due to heterogeneity identified. ^2^ downgraded due to suspected publication bias.

## References

[1] Roth G.A., Abate D., Abate K.H., Abay S.M., Abbafati C.N., Abbasi N., GBD 2017 Causes of Death Collaborators (2018). Global, Regional, and National Age-Sex-Specific Mortality for 282 Causes of Death in 195 Countries and Territories, 1980–2017: A Systematic Analysis for the Global Burden of Disease Study 2017. Lancet.

[2] Roth G.A., Johnson C., Abajobir A., Abd-Allah F., Abera S.F., Abyu G., Ahmed M., Aksut B., Alam T., Alam K. (2017). Global, Regional, and National Burden of Cardiovascular Diseases for 10 Causes, 1990 to 2015. J. Am. Coll. Cardiol..

[3] WHO (2019). CVD Risk Chart Working Group World Health Organization Cardiovascular Disease Risk Charts: Revised Models to Estimate Risk in 21 Global Regions. Lancet Glob. Health.

[4] Gaziano T.A., Abrahams-Gessel S., Alam S., Alam D., Ali M., Bloomfield G., Carrillo-Larco R.M., Dorairaj P., Gutierrez L., Irazola V. (2016). Comparison of Nonblood-Based and Blood-Based Total CV Risk Scores in Global Populations. Glob. Heart.

[5] Beck J.D., Pankow J., Tyroler H.A., Offenbacher S. (1999). Dental Infections and Atherosclerosis. Am. Heart J..

[6] Beck J.D., Slade G., Offenbacher S. (2000). Oral Disease, Cardiovascular Disease and Systemic Inflammation. Periodontology.

[7] Beck J.D., Offenbacher S. (2001). The Association between Periodontal Diseases and Cardiovascular Diseases: A State-of-the-Science Review. Ann. Periodontol..

[8] Sanz M., Del Castillo A.M., Jepsen S., Gonzalez-Juanatey J.R., D’Aiuto F., Bouchard P., Chapple I., Dietrich T., Gotsman I., Graziani F. (2020). Periodontitis and Cardiovascular Diseases. Consensus Report. Glob. Heart.

[9] Tomás I., Diz P., Tobías A., Scully C., Donos N. (2012). Periodontal Health Status and Bacteraemia from Daily Oral Activities: Systematic Review/Meta-Analysis. J. Clin. Periodontol..

[10] Balejo R.D.P., Cortelli J.R., Costa F.O., Cyrino R.M., Aquino D.R., Cogo-Müller K., Miranda T.B., Moura S.P., Cortelli S.C. (2017). Effects of Chlorhexidine Preprocedural Rinse on Bacteremia in Periodontal Patients: A Randomized Clinical Trial. J. Appl. Oral Sci..

[11] Rafferty B., Jönsson D., Kalachikov S., Demmer R.T., Nowygrod R., Elkind M.S.V., Bush H., Kozarov E. (2011). Impact of Monocytic Cells on Recovery of Uncultivable Bacteria from Atherosclerotic Lesions. J. Intern. Med..

[12] Kakehashi S., Stanley H.R., Fitzgerald R.J. (1965). The Effects of Surgical Exposures of Dental Pulps in Germ-Free and Conventional Laboratory Rats. Oral Surg. Oral Med. Oral Pathol..

[13] Cotti E., Dessì C., Piras A., Mercuro G. (2011). Can a Chronic Dental Infection Be Considered a Cause of Cardiovascular Disease? A Review of the Literature. Int. J. Cardiol..

[14] Caplan D.J., Chasen J.B., Krall E.A., Cai J., Kang S., Garcia R.I., Offenbacher S., Beck J.D. (2006). Lesions of Endodontic Origin and Risk of Coronary Heart Disease. J. Dent. Res..

[15] An G.K., Morse D.E., Kunin M., Goldberger R.S., Psoter W.J. (2016). Association of Radiographically Diagnosed Apical Periodontitis and Cardiovascular Disease: A Hospital Records–Based Study. J. Endod..

[16] Chauhan N., Mittal S., Tewari S., Sen J., Laller K. (2019). Association of Apical Periodontitis with Cardiovascular Disease via Noninvasive Assessment of Endothelial Function and Subclinical Atherosclerosis. J. Endod..

[17] Jakovljevic A., Duncan H.F., Nagendrababu V., Jacimovic J., Milasin J., Dummer P.M.H. (2020). Association between Cardiovascular Diseases and Apical Periodontitis: An Umbrella Review. Int. Endod. J..

[18] Messing M., Souza L.C.d., Cavalla F., Kookal K.K., Rizzo G., Walji M., Silva R., Letra A. (2019). Investigating Potential Correlations between Endodontic Pathology and Cardiovascular Diseases Using Epidemiological and Genetic Approaches. J. Endod..

[19] Cowan L.T., Lakshminarayan K., Lutsey P.L., Beck J., Offenbacher S., Pankow J.S. (2020). Endodontic Therapy and Incident Cardiovascular Disease: The Atherosclerosis Risk in Communities (ARIC) Study. J. Public Health Dent..

[20] Koletsi D., Iliadi A., Tzanetakis G., Vavuranakis M., Eliades T. (2020). Cardiovascular Disease and Chronic Dental Infection. Is There an Association? A Systematic Review and Meta-Analysis Protocol [osf.io/mxa24].

[21] Stroup D.F., Berlin J.A., Morton S.C., Olkin I., Williamson G.D., Rennie D., Moher D., Becker B.J., Sipe T.A., Thacker S.B. (2000). Meta-Analysis of Observational Studies in Epidemiology: A Proposal for Reporting. Meta-Analysis Of Observational Studies in Epidemiology (MOOSE) Group. JAMA.

[22] Fleming P.S., Koletsi D., Pandis N. (2014). Blinded by PRISMA: Are Systematic Reviewers Focusing on PRISMA and Ignoring Other Guidelines?. PLoS ONE.

[23] Sterne J.A., Hernán M.A., Reeves B.C., Savović J., Berkman N.D., Viswanathan M., Henry D., Altman D.G., Ansari M.T., Boutron I. (2016). ROBINS-I: A Tool for Assessing Risk of Bias in Non-Randomised Studies of Interventions. BMJ.

[24] Egger M., Davey Smith G., Schneider M., Minder C. (1997). Bias in Meta-Analysis Detected by a Simple, Graphical Test. BMJ.

[25] Guyatt G.H., Oxman A.D., Vist G.E., Kunz R., Falck-Ytter Y., Alonso-Coello P., Schünemann H.J., GRADE Working Group (2008). GRADE: An Emerging Consensus on Rating Quality of Evidence and Strength of Recommendations. BMJ.

[26] Balshem H., Helfand M., Schünemann H.J., Oxman A.D., Kunz R., Brozek J., Vist G.E., Falck-Ytter Y., Meerpohl J., Norris S. (2011). GRADE Guidelines: 3. Rating the Quality of Evidence. J. Clin. Epidemiol..

[27] Costa T.H.R., Neto J.A.d.F., de Oliveira A.E.F., e Maia M.D.F.L., de Almeida A.L. (2014). Association between Chronic Apical Periodontitis and Coronary Artery Disease. J. Endod..

[28] de Oliveira B.P., Cruz Câmara A., Aguiar M. (2017). Prevalence of Asymptomatic Apical Periodontitis and its Association with Coronary Artery Disease in a Brazilian Subpopulation. Acta Stomatol. Croat..

[29] Frisk F., Hakeberg M., Ahlqwist M., Bengtsson C. (2003). Endodontic Variables and Coronary Heart Disease. Acta Odontol. Scand..

[30] Garrido M., Cárdenas A.M., Astorga J., Quinlan F., Valdés M., Chaparro A., Carvajal P., Pussinen P., Huamán-Chipana P., Jalil J.E. (2019). Elevated Systemic Inflammatory Burden and Cardiovascular Risk in Young Adults with Endodontic Apical Lesions. J. Endod..

[31] Gomes M.S., Hugo F.N., Hilgert J.B., Sant’Ana Filho M., Padilha D.M.P., Simonsick E.M., Ferrucci L., Reynolds M.A. (2016). Apical Periodontitis and Incident Cardiovascular Events in the Baltimore Longitudinal Study of Ageing. Int. Endod. J..

[32] Jansson L., Lavstedt S., Frithiof L., Theobald H. (2001). Relationship between Oral Health and Mortality in Cardiovascular Diseases: Oral Health and Mortality. J. Clin. Periodontol..

[33] Joshipura K., Pitiphat W., Hung H., Willett W., Colditz G., Douglass C. (2006). Pulpal Inflammation and Incidence of Coronary Heart Disease. J. Endod..

[34] Liljestrand J.M., Mäntylä P., Paju S., Buhlin K., Kopra K.A.E., Persson G.R., Hernandez M., Nieminen M.S., Sinisalo J., Tjäderhane L. (2016). Association of Endodontic Lesions with Coronary Artery Disease. J. Dent. Res..

[35] Lin P.-Y., Chien K.-L., Chang H.-J., Chi L.-Y. (2015). Unfinished Root Canal Treatments and the Risk of Cardiovascular Disease. J. Endod..

[36] Pasqualini D., Bergandi L., Palumbo L., Borraccino A., Dambra V., Alovisi M., Migliaretti G., Ferraro G., Ghigo D., Bergerone S. (2012). Association among Oral Health, Apical Periodontitis, CD14 Polymorphisms, and Coronary Heart Disease in Middle-Aged Adults. J. Endod..

[37] Petersen J., Glaßl E.-M., Nasseri P., Crismani A., Luger A.K., Schoenherr E., Bertl K., Glodny B. (2014). The Association of Chronic Apical Periodontitis and Endodontic Therapy with Atherosclerosis. Clin. Oral Investig..

[38] Segura-Egea J.J., Jimenez-Moreno E., Calvo-Monroy C., Ríos-Santos J.V., Velasco-Ortega E., Sánchez-Domínguez B., Castellanos-Cosano L., Llamas-Carreras J.M. (2010). Hypertension and Dental Periapical Condition. J. Endod..

[39] Virtanen E., Nurmi T., Söder P.-Ö., Airila-Månsson S., Söder B., Meurman J.H. (2017). Apical Periodontitis Associates with Cardiovascular Diseases: A Cross-Sectional Study from Sweden. BMC Oral Health.

[40] Willershausen B., Kasaj A., Willershausen I., Zahorka D., Briseño B., Blettner M., Genth-Zotz S., Münzel T. (2009). Association between Chronic Dental Infection and Acute Myocardial Infarction. J. Endod..

[41] Willershausen I., Weyer V., Peter M., Weichert C., Kasaj A., Münzel T., Willershausen B. (2014). Association between Chronic Periodontal and Apical Inflammation and Acute Myocardial Infarction. Odontology.

[42] Friedlander A.H., Sung E.C., Chung E.M., Garrett N.R. (2010). Radiographic Quantification of Chronic Dental Infection and Its Relationship to the Atherosclerotic Process in the Carotid Arteries. Oral Surg. Oral Med. Oral Pathol. Oral Radiol. Endod..

[43] Vasan R.S., Benjamin E.J. (2016). The Future of Cardiovascular Epidemiology. Circulation.

[44] Unudurthi S.D., Luthra P., Bose R.J.C., McCarthy J.R., Kontaridis M.I. (2020). Cardiac Inflammation in COVID-19: Lessons from Heart Failure. Life Sci..

[45] World Health Organization (WHO) Cardiovascular diseases. https://www.Who.Int/Cardiovascular_diseases/About_cvd/En/.

[46] Wang L.C., Gu A.Q., Sun C.L., Xu H., Ni X.S., Wang R.J., Zhao X.Y., Wang Q.C. (2020). Cross-Sectional Study of Factors Correlated to Quality of Life in Patients with Coronary Artery Disease and Diabetic Retinopathy. J. Biol. Regul. Homeost. Agents.

[47] Li S., Tang X., Luo Y., Wu B., Huang Z., Li Z., Peng L., Ling Y., Zhu J., Zhong J. (2020). Impact of Long-Term Glucose Variability on Coronary Atherosclerosis Progression in Patients with Type 2 Diabetes: A 2.3 Year Follow-up Study. Cardiovasc. Diabetol..

[48] Parada-Turska J., Wójcicka G., Beltowski J. (2020). Paraoxonase 1 Phenotype and Protein N-Homocysteinylation in Patients with Rheumatoid Arthritis: Implications for Cardiovascular Disease. Antioxidants.

[49] Zafar R. (2015). An Insight into Pathogenesis of Cardiovascular Diseases. J. Integr. Cardiol..

[50] Koivisto T., Bowles W.R., Rohrer M. (2012). Frequency and Distribution of Radiolucent Jaw Lesions: A Retrospective Analysis of 9,723 Cases. J. Endod..

[51] Braz-Silva P.H., Bergamini M.L., Mardegan A.P., De Rosa C.S., Hasseus B., Jonasson P. (2019). Inflammatory Profile of Chronic Apical Periodontitis: A Literature Review. Acta Odontol. Scand..

[52] de Oliveira Rodini C., Batista A.C., Lara V.S. (2004). Comparative Immunohistochemical Study of the Presence of Mast Cells in Apical Granulomas and Periapical Cysts: Possible Role of Mast Cells in the Course of Human Periapical Lesions. Oral Surg. Oral Med. Oral Pathol. Oral Radiol. Endod..

[53] Aranha A.M.F., Repeke C.E., Garlet T.P., Vieira A.E., Campanelli A.P., Trombone A.P.F., Letra A., Silva R.M., Garlet G.P. (2013). Evidence Supporting a Protective Role for Th9 and Th22 Cytokines in Human and Experimental Periapical Lesions. J. Endod..

[54] Bergandi L., Giuggia B., Alovisi M., Comba A., Silvagno F., Maule M., Aldieri E., Scotti N., Scacciatella P., Conrotto F. (2019). Endothelial Dysfunction Marker Variation in Young Adults with Chronic Apical Periodontitis before and after Endodontic Treatment. J. Endod..

[55] Munz M., Richter G.M., Loos B.G., Jepsen S., Divaris K., Offenbacher S., Teumer A., Holtfreter B., Kocher T., Bruckmann C. (2018). Genome-Wide Association Meta-Analysis of Coronary Artery Disease and Periodontitis Reveals a Novel Shared Risk Locus. Sci. Rep..

[56] Aminoshariae A., Kulild J.C., Fouad A.F. (2018). The Impact of Endodontic Infections on the Pathogenesis of Cardiovascular Disease(s): A Systematic Review with Meta-Analysis Using GRADE. J. Endod..

[57] Berlin-Broner Y., Febbraio M., Levin L. (2017). Association between Apical Periodontitis and Cardiovascular Diseases: A Systematic Review of the Literature. Int. Endod. J..

[58] Khalighinejad N., Aminoshariae M.R., Aminoshariae A., Kulild J.C., Mickel A., Fouad A.F. (2016). Association between Systemic Diseases and Apical Periodontitis. J. Endod..

[59] González Navarro B., Pintó Sala X., Jané Salas E. (2017). Relationship between Cardiovascular Disease and Dental Pathology. Systematic Review. Med. Clin..

